# Big or fast: two strategies in the developmental control of body size

**DOI:** 10.1186/s12915-015-0173-x

**Published:** 2015-08-04

**Authors:** H. Frederik Nijhout

**Affiliations:** Department of Biology, Duke University, Durham, NC 27708 USA

## Abstract

Adult body size is controlled by the mechanisms that stop growth when a species-characteristic size has been reached. The mechanisms by which size is sensed and by which this information is transduced to the growth regulating system are beginning to be understood in a few species of insects. Two rather different strategies for control have been discovered; one favors large body size and the other favors rapid development.

## Commentary

Age and size at maturity are arguably the two most important life history traits of animals. Variation in both traits has severe effects on fitness: age at maturity affects generation time and size at maturity has a strong effect on reproductive capacity. Accordingly, age and size at maturity have been intensively studied from an evolutionary and ecological perspective [1, 2]. Yet in spite of their obvious importance, the genetic, developmental and physiological mechanisms that control age and size at maturity are for the most part unknown. Nutrition and hormones play obvious and well-established roles in growth, but the natural mechanisms that cause the cessation of growth when an animal reaches a species-specific size remain among the great puzzles in biology.

The recent paper by Hatem et al. [3] has shed new light on the developmental and physiological mechanisms that regulate growth and body size, and at the same time revealed the cause of a puzzling and troublesome difference in mechanism between the two species, *Manduca sexta* and *Drosophila melanogaster*, in which the control of size has been best studied.

Growing to a species-specific size requires a mechanism that can monitor size, and a response mechanism that stops growth and that is triggered when a particular size is reached. Such size-monitoring and response mechanisms are not known in any mammal, but they are now beginning to be well-understood in several insects, specifically *M. sexta*, a moth, and *D. melanogaster*, a fly. The proximate trigger for the cessation of growth in insects is well-known, namely a pulse of the steroid hormone ecdysone that occurs at the end of larval life. This pulse causes the animal to stop feeding and begin the metamorphic molt. Insects do not grow as adults, so the size of the larva at the time of this ecdysone pulse fully determines the size of the adult insect.

## How do you know how big you are?

The control of the timing of this ecdysone pulse was first investigated in *Manduca* [4, 5]. About half way through the fifth and final larval instar, larvae of *Manduca* pass what has been called the critical weight. This is the size at which the physiological events that lead to the secretion of ecdysone are set in motion. During this instar the secretion of ecdysone is repressed by juvenile hormone (JH), and at the critical weight the secretion of JH stops and expression of the enzyme JH-esterase (the principal catabolic enzyme for JH) is upregulated. Suppression of ecdysone secretion is relieved over a period of 24–60 hours (depending on the genetic background). Thus, the final size of the larva is set by the value of the critical weight and the duration of the period during which JH is eliminated, called the terminal growth phase (TGP).

The size-monitoring mechanism that senses the critical weight was quite unexpected, and involves oxygen limitation by a non-growing tracheal system that becomes unable to keep up with the ever-increasing oxygen demands of a growing body. The size at which oxygen becomes limiting corresponds to the critical weight [6]. Rearing *Manduca* larvae under hypoxia lowers their critical weight and also lowers the size at which ecdysone is secreted and growth stops. A similar mechanism operates in *Drosophila* where larvae reared under hypoxia likewise yield miniature adult flies [7], presumably because the metamorphic molt is triggered prematurely. In both species the first step in the cascade that controls body size is oxygen restriction. In *Manduca* oxygen limitation defines the critical weight and triggers the decline in JH. In *Drosophila* there is no role for JH in regulating ecdysone secretion.

One of the most striking differences between *Manduca* and *Drosophila* is that *Drosophila* apparently has little or no JH in the third and final larval stage. And since in *Manduca* JH plays the key role in regulating body size, it was natural to assume that in *Drosophila* control of size must be quite different. This idea was enhanced by the discovery that interference with insulin signaling could have dramatic effects on adult body size in *Drosophila*. Suppression of insulin production or inactivation of the insulin receptor or receptor substrate resulted in miniature flies [8], whereas overexpression of insulin resulted in giant flies [9].

Another interesting difference between *Manduca* and *Drosophila* is their response to starvation after passing the critical weight. When *Manduca* larvae are starved after they have passed the critical weight their time to ecdysone secretion and pupation is identical to that of larvae that continue to feed normally [5]. By contrast, when *Drosophila* larvae are starved their time to pupariation is accelerated [10]. This premature metamorphosis has been called the bailout mechanism [11]. It is a common adaptation in animals that live in evanescent habitats where finding a new source of food after the current one runs out is not possible, though the mechanism of the bailout response is still unknown.

## All species are not the same

Because of these differences in the endocrine biology of the two species, work on the regulation of size in *Manduca* has focused mostly on the physiology of the critical weight and the role of JH in controlling ecdysone secretion, while in *Drosophila* most work has focused on the role of insulin signaling and the relative roles of members of the insulin signaling network such as FOXO and TOR [12].

Although many aspects of the morphology and physiology of *Drosophila* are highly derived compared with other insects, it seemed difficult to understand why *Drosophila* would have evolved such a radically different control mechanisms over body size than what was found in *Manduca*. This puzzle might be resolved if there were only a way to make *Drosophila* more like *Manduca*, or *Manduca* more like *Drosophila*.

It is the latter that was accomplished in the paper by Hatem et al. [3]. It turns out that there is a genetic strain of *Manduca*, the black strain, that, besides having black larvae and a much smaller adult body size, has a reduced level of JH during the larval stage [13]. In this latter respect it resembles *Drosophila*.

What Hatem et al. [3] did was to feed wild-type and black larvae on a diet containing rapamycin, which inhibits TOR signaling, one of the terminal steps in the insulin pathway. They found that although growth was slowed down in both strains, as one might expect, the wild-type strain eventually grew to a normal size whereas the black strain grew much larger than normal. Interestingly, the critical weight was not altered in either strain when fed rapamycin. The increase in size in black larvae must therefore have been due to a lengthening of the TGP.

**Fig. 1:**
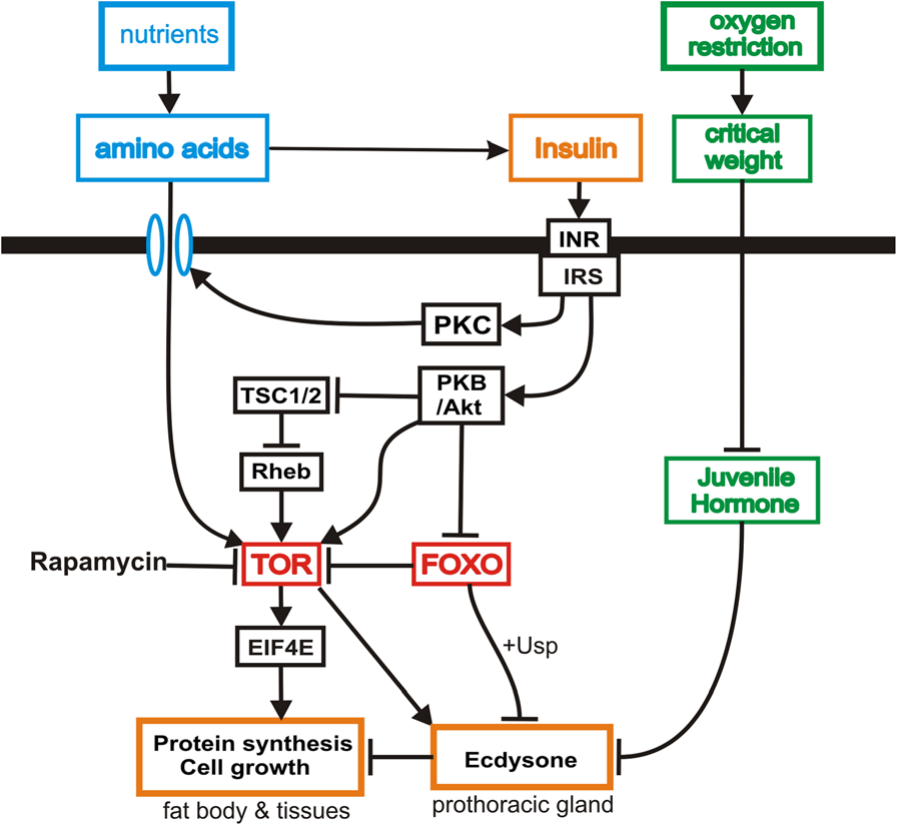
The insulin signaling network that links nutrition to growth and size. Nutrition in the form of amino acids stimulates insulin synthesis. Activation of the insulin receptor and receptor substrate (INR/INS) activates PKC and PKB/Akt via a multi-step pathway. PKC stimulates uptake of amino acids, which activate TOR. Insulin also activates TOR via PKB/Akt. TOR is inhibited by FOXO, which is inhibited by PKB/Akt. TOR stimulates protein synthesis via EIF4E transcriptional initiator, and causes cellular growth in tissues. In the prothoracic glands TOR stimulates ecdysone synthesis. Ecdysone is inhibited by FOXO when bound to Ultraspiracle (Usp) [16]. Inhibition of TOR by rapamycin inhibits growth and ecdysone synthesis. Ecdysone is also inhibited by juvenile hormone, which begins to decay at the critical weight, due to oxygen restriction. Thus, the ultimate control in this network resides in two environmental factors, nutrition and oxygen restriction

TOR not only controls amino acid sensing, protein synthesis and cellular growth in tissues, but also ecdysone biosynthesis in the prothoracic glands (Fig. 1). The delay in molting was presumably due to the fact that interference with TOR also interfered with and delayed ecdysone secretion. Hatem et al. [3] suggest that rapamycin did not have this effect in the wild-type strain because its effect is overridden by the high JH levels in this strain.

Another interesting property of the black strain is that when larvae that have been fed rapamycin are starved, their time to metamorphosis is accelerated, just as is observed in the bail-out response in *Drosophila*, but which does not happen in the wild-type strain of *Manduca*. Thus, in several respects the developmental physiology of the black strain is more like that of *Drosophila* than that of wild-type *Manduca*.

## Two different strategies

**Fig. 2:**
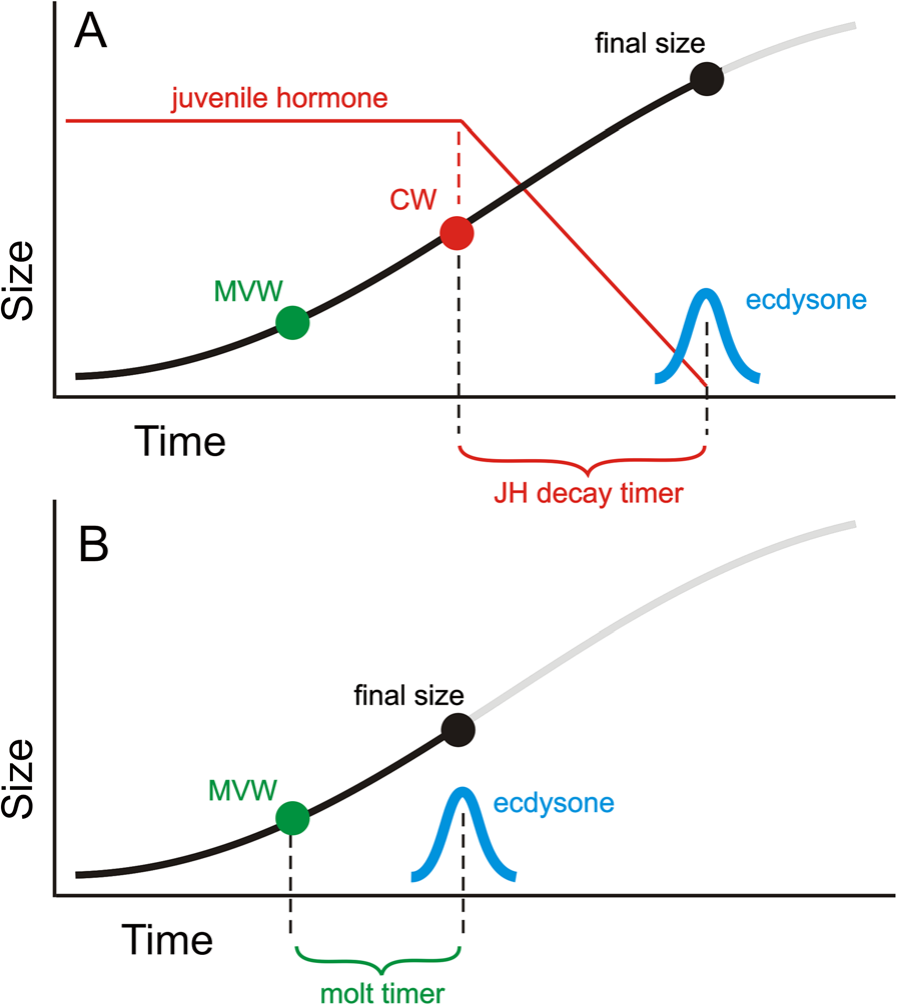
Alternative control mechanism for body size. Sigmoidal growth curves with two different mechanisms that stop growth. **a** In the presence of juvenile hormone (JH), as in *Manduca*, ecdysone secretion cannot occur until JH has disappeared. The decline of JH begins when larvae pass the critical weight (CW). **b** In the absence of JH, as in *Drosophila*, ecdysone secretion follows attainment of the minimum viable weight (MVW) after a delay called the molt timer, whose mechanism is not yet understood. This leads to an earlier cessation of growth and a smaller body size than occurs when JH overrides the MVW mechanism

Hatem et al. [3] suggest that there must be two levels of control over ecdysone. The first one is primarily nutrition-sensitive and mediated by insulin/TOR signaling, and is triggered at the minimum viable weight (MVW), which starts a delay timer (whose mechanism is still unknown) that leads to ecdysone secretion [14]. This is the mechanism that operates in *Drosophila*. The second occurs in *Manduca*, where JH overrides the insulin/TOR-mediated mechanism. *Manduca* also has a MVW that lies much below the critical weight [15] and does not operate except under stressful conditions [14]. Normally JH decline begins at the critical weight and another delay timer (in this case the time required to eliminate JH) is triggered that eventually results in the secretion of ecdysone (Fig. 2).

It is interesting to speculate which of these control systems is primitive. Since the JH sensitivity and the critical weight occur later in development than the nutrition-sensitive control and the MVW, it looks like it might have a later evolutionary origin. Alternatively, it could be that, driven by the adaptive significance of being able to bail out of a deteriorating environment, *Drosophila* abandoned control by JH.

Hatem et al. [3] suggest that the JH-controlled mechanism favors large body size, but at the cost of more prolonged development, whereas eliminating the repression of ecdysone secretion by JH favors rapid development time. Thus, the differences between the control mechanisms in *Manduca* and *Drosophila* are most likely adaptations for very different life histories.

Which of these strategies is likely to be the ultimate cause of the difference remains to be elucidated. In the meantime, Hatem et al. [3] have taken a significant step in resolving the puzzling differences between *Manduca* and *Drosophila*, and this work lays the foundation for novel experimental approaches to uncover more fully the details of the developmental physiology of body size regulation.
